# Multi-omics sequencing provides insight into floral transition in *Catalpa bungei. C.A. Mey*

**DOI:** 10.1186/s12864-020-06918-y

**Published:** 2020-07-22

**Authors:** Zhi Wang, Wenjun Ma, Tianqing Zhu, Nan Lu, Fangqun Ouyang, Nan Wang, Guijuan Yang, Lisheng Kong, Guanzheng Qu, Shougong Zhang, Junhui Wang

**Affiliations:** 1 State Key Laboratory of Tree Genetics and Breeding, Key Laboratory of Tree Breeding and Cultivation of State Forestry Administration, Research Institute of Forestry, Chinese Academy of Forestry, Beijing, 100091 PR China; 2 Department of Biology Centre for Forest Biology, University of Victoria, Victoria, BC 11 Canada; 3 State Key Laboratory of Tree Genetics and Breeding (Northeast Forestry University), 26 Hexing Road, Harbin, 150040 PR China

**Keywords:** Floral transition, RNA sequencing, WGCNA, Early flowering, *Catalpa bungei*

## Abstract

**Background:**

Floral transition plays an important role in development, and proper time is necessary to improve the value of valuable ornamental trees. The molecular mechanisms of floral transition remain unknown in perennial woody plants. “Bairihua” is a type of *C. bungei* that can undergo floral transition in the first planting year.

**Results:**

Here, we combined short-read next-generation sequencing (NGS) and single-molecule real-time (SMRT) sequencing to provide a more complete view of transcriptome regulation during floral transition in *C. bungei*. The circadian rhythm-plant pathway may be the critical pathway during floral transition in early flowering (EF) *C. bungei*, according to horizontal and vertical analysis in EF and normal flowering (NF) *C. bungei*. SBP and MIKC-MADS-box were seemingly involved in EF during floral transition. A total of 61 hub genes were associated with floral transition in the MEturquoise model with Weighted Gene Co-expression Network Analysis (WGCNA). The results reveal that ten hub genes had a close connection with the *GASA homologue gene* (Cbu.gene.18280), and the ten co-expressed genes belong to five flowering-related pathways. Furthermore, our study provides new insights into the complexity and regulation of alternative splicing (AS). The ratio or number of isoforms of some floral transition-related genes is different in different periods or in different sub-genomes.

**Conclusions:**

Our results will be a useful reference for the study of floral transition in other perennial woody plants. Further molecular investigations are needed to verify our sequencing data.

## Background

Floral transition is the developmental process by which a plant transitions from vegetative growth to reproductive growth. During this process, inflorescence primordia instead of leaf primordia develop from the shoot apical meristem (SAM) [1–3]. Great progress has been made in understanding the factors that trigger floral transition [4]. A set of floral transition-related genes, such as *SPL* (*Squamosa-promoter binding protein-like*) [5–7], *TOC* (*Timing of cab expression 1*) [8], *LUX* (*Luxarrhythmo*) [8], *PIF* (*Phytochrome interacting factor*) [9], *CO* (*constans*) [10], *FRI* (*Frigida*) [11], *GA20ox* (*GA20oxidases*) [7], *GA3ox* (*GA3oxidases*) [12], *SOC1* (*Suppressor of overexpression of constans 1*) [13], have been detected, in addition to others [14, 15]. These genes are mainly categorized into five major pathways that regulate floral transition, including the age pathway, photoperiod and circadian clock pathway, autonomous pathway, vernalisation pathway and GA pathway [4]. These genes are independent and closely related to each other, forming sophisticated gene regulatory networks (GRNs) [1, 16]. For example, *SPL* is involved in inducing the expression of flowering integrator genes, namely, *LEAFY* (*LFY*) and APETALA1 (*AP1*), thereby triggering flowering [17]. *TOC* and *LUX* are the critical genes in circadian rhythms pathway [18, 19]. In term of the feed-back loop, *TOC1* can either directly or indirectly regulate *CCA1* and *LHY*, which in turn suppress *TOC1* expression by binding to its regulatory region [20, 21]. The circadian clock gene *LUX* affects flowering by forming the evening complex (EC) with *EARLY FLOWERING 3* (*ELF3*) and *ELF* [22]. *FRI* controls flowering by regulating the expression of the floral transition of floral repressor *FLC*, which encodes a MADS-box protein [11]. *CO* promotes flowering by directly activating the expression of its downstream genes including *FT* and *SOC1* [23]. *SOC1* is also regulated by active GA in the gibberellin pathway and positively regulated by *SPL* in the age pathway [24]. However, most of these studies were focused on annual herbaceous model plants, such as *Arabidopsis* [25] and Rice [26]. In perennial woody plants, the studies involved in floral transition are still in their infancy [27, 28]. Few studies have been conducted on floral transition in trees, partly due to the long juvenile phase and the difficulty in distinguishing vegetative buds from flowering buds at the beginning of the budding phase of trees. *Catalpa bungei. C.A. Mey* (*C. bungei*, Family: Bignoniaceae) is an important ornamental tree species in China [29, 30]. *C. bungei* not only has good woody properties but is also famous for its beautiful flowers. The commercial value of this species is largely related to its flowering time. The optimum flowering time greatly affects the quality of *C. bungei*. *C. bungei* is a perennial tree that undergoes its first floral transition in the fifth year or more of planting. However, an early flowering (EF), the new natural variety of *C. bungei*, was found to undergo floral transition in the first planting year, and almost 100% of its buds were mixed buds, which is very rare for woody plants (http://www.forestry.gov.cn/). At present, the research on *C. bungei* mainly focuses on the development of wood and flower organs [29–32], and the study of the flowering of *C. bungei* is just beginning. The EF variety, which only develops mixed buds, solves the problem of material selection and provides an opportunity to evaluate the floral transition process in perennial ornamental woody plants*.*

Next-generation sequencing (NGS) technologies have become a powerful tool for describing gene expression levels. However, NGS is limited by the necessity of short reads during library construction [33]. Single-molecule real-time (SMRT) sequencing technology overcomes this limitation by generating kilobase-sized sequencing reads [34]. The combination of NGS and SMRT approaches not only enables the overall transcript level of each gene to be analysed but also provides vital insight into alternative splicing (AS) events [35], which have fundamental roles in a wide range of plant growth and development processes [36–41]. In particular, the AS of genes, such as *FT, FLC*, and *PRR,* regulates floral transition [19, 20, 40, 42–46].

The NGS and SMRT sequencing platform was used to further investigate the genes involved in floral transition. In this study, we analysed the data from three perspectives, namely, horizontal analysis, vertical analysis and WGCNA. A total of 61 hub genes that may be associated with floral transition in *C. bungei* were mined. Several potential protein interactions were found by regulatory network analysis. The complexity of AS events in the EF and NF varieties was addressed via SMRT sequencing. More than 50% of the identified genes had multiple structures. This work provides a guideline for future studies on how woody plants regulate the expression of key genes during floral transition.

## Results

### Grouping of the buds from EF variety and NF variety

**Fig. 1:**
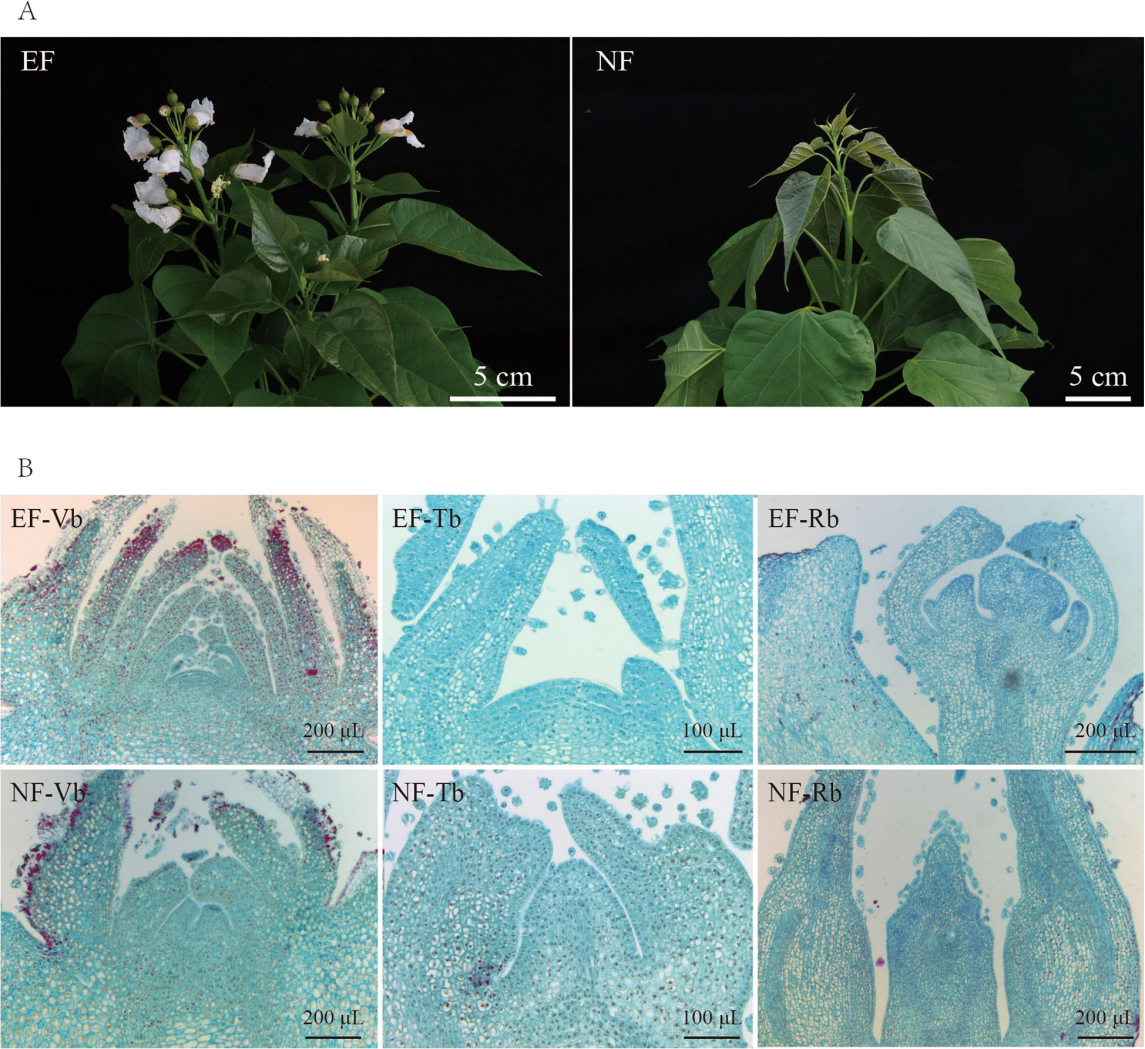
Photos and internal morphology of the EF and NF buds in the first planting year. **a** Photos of the EF and NF buds in the first planting year. Pictures are the EF phenotype (top) and NF phenotype (top). Early flowering (EF), Normal flowering (NF)*.***b** Internal morphology of the EF and NF buds. Sections of the buds from the EF and NF varieties. EF-Vb, photo of the vegetative buds from the EF variety; EF-Tb, photo of the transition buds from the EF variety; EF-Rb, photo of the reproduction buds from the EF variety; NF-Vb, photo of the vegetative buds from the NF variety; NF-Tb, photo of the transition buds from the NF variety; NF-Rb, photo of the reproduction buds from the NF variety. Vegetative buds (Vb), transition buds (Tb), and reproductive buds (Rb). Early flowering (EF), Normal flowering (NF)

An EF variety was used to study floral transition. A NF variety was used as a control (Fig. 1a). The EF buds were subgrouped into three consecutive differentiation stages, namely, vegetative buds (Vb), transition buds (Tb), and reproductive buds (Rb), according to their anatomical structure (Fig. 1b). In the Vb, the reproductive shoot apex was still invisible. In the Tb, the reproductive shoot apexes had initiated. In the Rb, the development of the reproductive shoot apex had completed, and the differentiated sepals, petals, pistils, etc. were observed. The NF buds were always Vb morphologically. However, we subgrouped them artificially into the three stages according to the corresponding collection date for the control. Since the molecular regulation of floral transition begins far before morphological changes occur, many critical molecular regulations should have already occurred in the Vb [29, 31, 47].

### Illumina-based RNA and SMRT sequencing and assembly

To explore the molecular regulation during floral transition in *C. bungei*, we carried out NGS and SMRT sequencing for the stem apical buds. The stem apical buds (Vb, Tb and Rb) from the EF and NF varieties were prepared for NGS. Each group had three biological replicates. A total of 18 mRNA samples were subjected to 2*150 bp paired-end sequencing using the HiSeq 4000 platform, which produced more than 13G of clean reads (Table S1). Subsequently, the RNA samples were pooled according to EF and NF for SMRT sequencing. The full-length cDNAs of these samples were sequenced and constructed using the PacBio RS II platform. In total, 13 SMRT cells and 16 SMRT cells were used for the EF and NF mixed samples, respectively, with three size fractions, namely, 1–2 kb, 2–3 kb, and > 3 kb. The mean ReadsOfInsert lengths produced in the EF and NF samples were 2702 bp and 4028 bp, respectively. ReadsOfInserts were composed of 261,651 full-length non-chimeric reads and 175,647 non-full-length reads in EF and 122,967 full-length reads and 339,065 non-full-length reads in NF. The average lengths of the full-length non-chimeric reads were 2592 bp and 2605 bp in EF and NF, respectively. The non-full-length transcripts and the full-length transcripts were classified based on the presence of 5′ primers, 3′ primers and poly(A) tails reaching near-saturation of gene discovery (Table S2, Fig. S1, Fig. S2). The transcript length distributions generated by these two platforms showed that approximately 88% of the assembled transcripts from the Illumina platform and 11% of the transcripts from the SMRT reads were < 600 bases (Fig. S3A)_._ A total of 22,934 annotated genes were detected by Illumina RNA-seq. In contrast, 14,753 EF and 15,212 NF annotated genes were detected by SMRT sequencing. Of the annotated genes, 11,631 genes were found by both Illumina and SMRT. A total of 6628 genes were identified only by Illumina, and 1450 genes were identified only by SMRT, i.e., 383 EF-specific genes, 489 NF-specific genes and 578 common genes in both the EF and NF varieties (Fig. S3B). The high sensitivity of SMRT makes it possible to detect the alternative polyadenylation (APA) in the transcriptome high-throughput data. In our experiment, of the 36,935 genes detected by SMRT, 13,843 transcripts had one poly (A) site, while 1962 genes had at least five poly (A) sites (Fig. S3C). These APAs could increase transcriptome complexities, subsequently affecting post-transcriptional regulation.

### Differential gene expression during floral transition

**Fig. 2:**
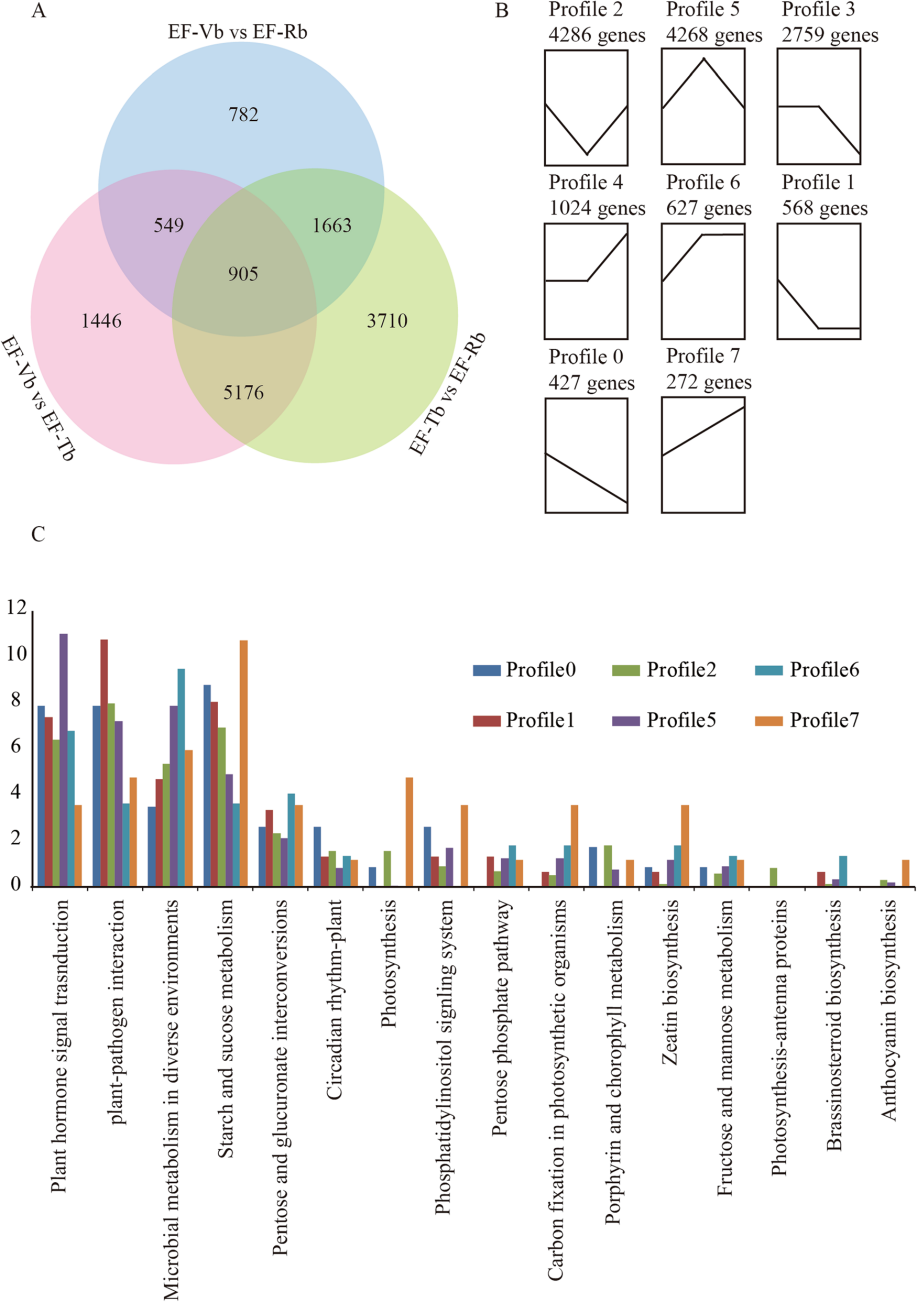
Analysis of differential gene expression during floral transition of the EF variety. **a** Venn diagram analysis of the number of DEGs between EF-Vb vs EF-Tb, EF-Tb vs EF-Rb and EF-Vb vs EF-Rb. EF-Vb, the data of vegetative buds from the EF variety; EF-Tb, the data of the transition buds from the EF variety; EF-Rb, the data of the reproduction buds from the EF variety. **b** The 8 significant expression profiles during floral transition of EF. **c** Partial KEGG pathways associated with floral transition of EF. The longitudinal axis represents the percent of the number of genes. The horizontal axis represents the pathway names. The dark blue rectangle indicates the data were from Profile 0. The red rectangle indicates the data were from Profile 1. The green rectangle indicates the data were from Profile 2. The purple rectangle indicates the data were from Profile 5. The light blue rectangle indicates the data were from Profile 6. The orange rectangle indicates the data were from Profile 7

To characterize the expression profiles of the 14,231 EF DEGs and 7378 NF DEGs, the expression data υ (from Vb to Tb and Tb to Rb) were normalized to 0, log_2_^(Tb/Vb)^, and log_2_^(Rb/Vb)^. In total, all the DEGs clustered into eight profiles based on STEM analysis (Fig. 2a and Fig. S4A). It was assumed that the DEGs obtained from the vertical analysis between EF-Vb and EF-Tb were mainly associated with floral transition. In our data, genes belonging to Profile 3 and Profile 4 showed no significant difference between EF-Vb and EF-Tb. Therefore, Profiles 0, 1, 2, 5, 6, and 7 were chosen for subsequent analyses (Fig. 2b). Profiles 0, 1, and 2 were downregulated between Vb and Tb in the EF buds and contained 427, 568 and 4286 DEGs, respectively. Profiles 5, 6, and 7 were upregulated between Vb and Tb in the EF buds and contained 4268, 627 and 272 DEGs, respectively.

All the DEGs in EF buds that belonged to profiles 0, 1, 2, 5, 6 and 7 were subjected to KEGG pathway enrichment analysis (Table S3). The KEGG pathways associated with plant floral transition are listed in Fig. 2c. Plant-pathogen interaction (ko04626), plant hormone signal transduction (ko04075), microbial metabolism in diverse environments (ko01120), starch and sucrose metabolism (ko00500) and circadian rhythm-plant (ko04712) were significantly enriched in all six profiles. Plant-pathogen interaction (ko04626) was significantly enriched in Profile 5, plant hormone signal transduction was significantly enriched in Profile 2, starch and sucrose metabolism was significantly enriched in Profile 7 and circadian rhythm-plant was significantly enriched in Profile 0. Most of the pathways, such as photosynthesis (ko00195), brassinosteroid biosynthesis, and anthocyanin biosynthesis, were not enriched in all six profiles. The photosynthesis and anthocyanin biosynthesis pathways were obviously enriched obviously in Profile 7. Brassinosteroid biosynthesis was obviously enriched in Profile 6. In addition, the photosynthesis-antenna proteins pathway was only enriched in Profile 2. The high expression of the circadian rhythm-plant pathway in EF-Vb implied that circadian rhythm-related genes may promote the activation of related downstream pathways, eventually leading to early flowering. In addition, the KEGG pathway enrichment results of DEGs in NF buds were mainly related to carbohydrate metabolism and energy metabolism, and no related plant floral transition pathways were found (Fig. S4B).

### Gene sets differentially expressed between the EF and NF buds

**Fig. 3:**
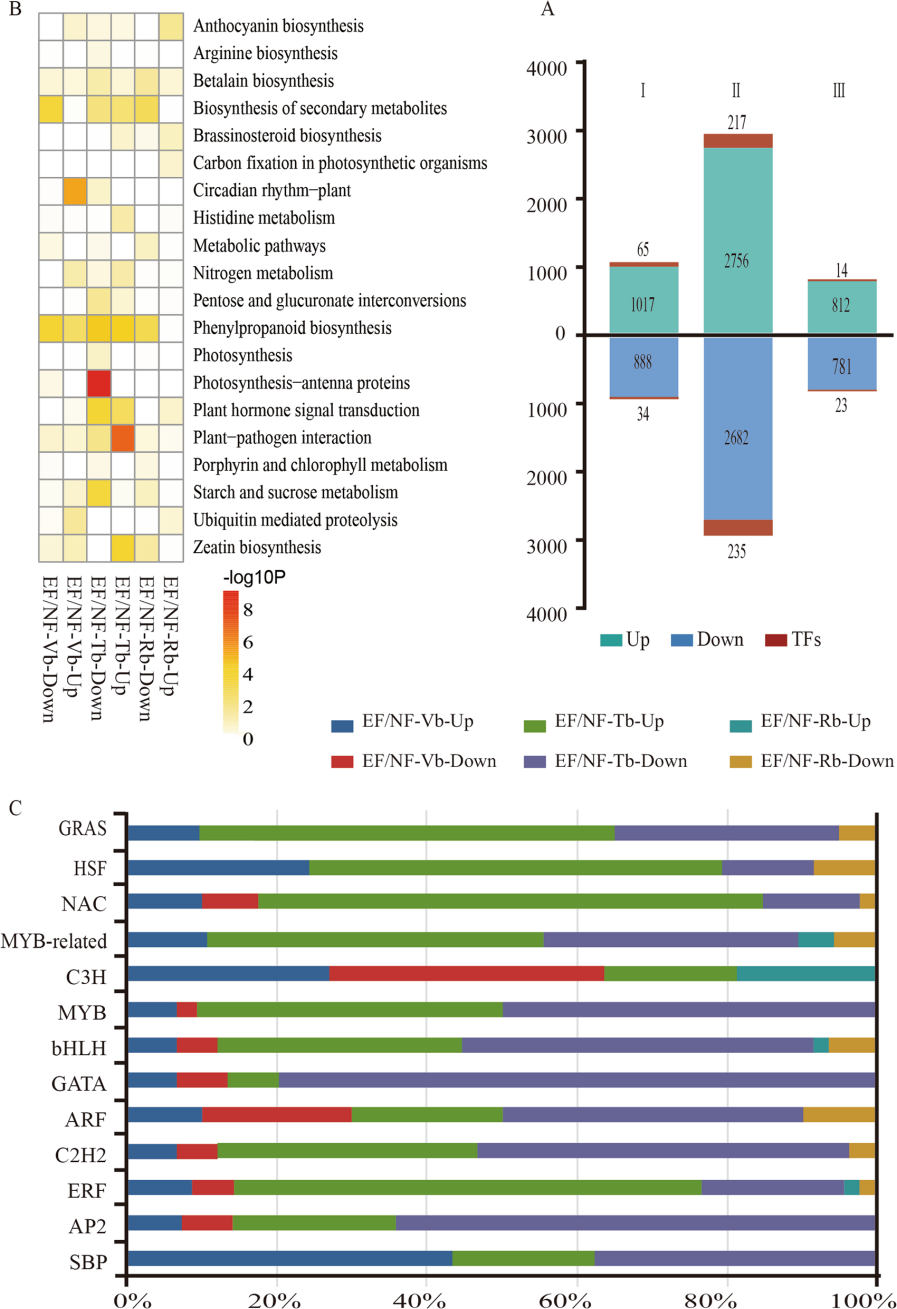
Differential gene expression in EF compared to NF at different stages of floral transition. **a** The number of upregulated (upper bars) and downregulated (lower bars) genes at each stage of floral transition in EF compared to NF is given. I indicates EF-Vb vs NF-Vb; II indicates EF-Tb vs NF-Tb; III indicates EF-Rb vs NF-Rb. The number of TFs up- or downregulated at each stage of floral transition is also given. EF-Vb, the number of genes of vegetative buds from the EF variety; EF-Tb, the number of genes of the transition buds from the EF variety; EF-Rb, the number of genes of the reproduction buds from the EF variety; NF-Vb, the number of genes of vegetative buds from the NF variety; NF-Tb, the number of genes of the transition buds from the NF variety; NF-Rb, the number of genes of the reproduction buds from the NF variety. **b** KEGG analysis of at different stages of floral transition in down- and upregulated genes in EF. The colour scale at the bottom represents significance (corrected *P*-value). **c** Partial TF families showing up-regulation or down-regulation at different stages during floral transition between EF and NF

To investigate the DEGs that might lead to floral transition, horizontal analysis was performed between EF and NF. In total, 4584 genes exhibited significantly higher expression and 4351 genes exhibited significantly lower expression at different stages in EF compared to NF. There were 1905 DEGs between EF-Vb and NF-Vb (including 65 upregulated and 34 downregulated TFs). There were 5438 DEGs between EF-Tb and NF-Tb (including 217 upregulated and 235 downregulated TFs). There were 1593 DEGs between EF-Rb and NF-Rb (including 14 upregulated and 23 downregulated TFs) (Fig. 3a).

TFs are critical for development transition in plants [48, 49]. In our data, 58 TF families were significantly differentially expressed in EF compared to NF during floral transition (Table S4). Thirteen of the 58 TF families, such as *B3* [50], *bHLH* [51], *GRAS* [52, 53], *ARF* [54], *AP2* [55], *SBP* [6] have been reported as important developmental regulators (Fig. 3b). *GRAS*, *HSF*, *NAC* and *MYB-related genes* showed significant enrichment in EF/NF-Tb-UP. *MYB*, *bHLH*, and *GATA* showed significant enrichment in EF/NF-Rb-UP. In addition, *C3H* and *SBP* showed significant enrichment in EF/NF-Vb. Furthermore, all *SBPs* were only enriched via upregulation in the EF compared to NF in vegetative buds. This implies that the SBP family might relate with the early floral transition in EF, similar to the function of SBP in other plants during floral transition [7, 31, 56–62].

The DEGs were assigned to 67 KEGG pathways. The top 20 pathways are presented in (Table S5). Enrichment analysis suggested circadian rhythm-plant (ko04712) and ubiquitin mediated proteolysis (ko04120) were significantly enriched in Vb, while photosynthesis-antenna proteins (ko00196), nitrogen metabolism (ko00910) and plant−pathogen interaction (ko04626) were significantly enriched in Tb (Fig. 3c). These results combined with data from the vertical analysis, further supported the idea that the circadian rhythm-plant pathway was critical during floral transition.

### Identification of conserved and/or divergent gene co-expression modules

WGCNA was performed to obtain a comprehensive understanding of genes expressed in the successive developmental stages of EF and NF and to identify the genes that might be associated with floral transition. After filtering out the genes with low expression (FPKM < 0.05), 34,483 genes were retained for WGCNA. Co-expression networks were constructed on the basis of pair-wise correlations of gene expression across all samples. Modules were defined as clusters of highly interconnected genes, and genes within the same cluster had high correlations.

**Fig. 4:**
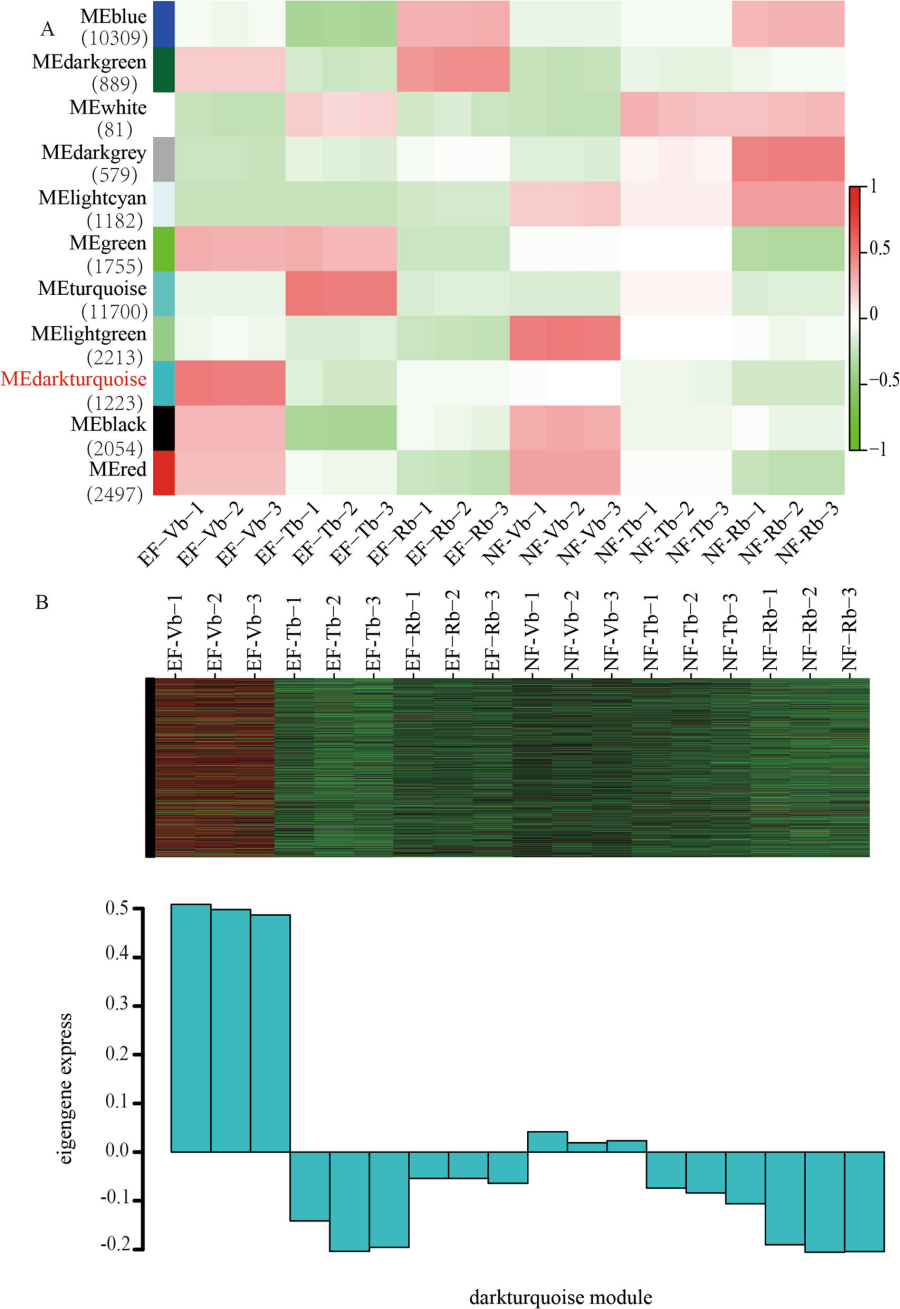
Co-expression networks during floral transition in EF and NF. **a** Module-trait associations were evaluated by correlations between MEs, and traits are shown. The left panel shows the 11 modules and the number of member genes. The colour scale on the right shows module-trait correlations from − 1 (Green) to 1 (Blue). EF-Vb represents the vegetative buds from early flowering, as a trait; EF-Tb represents the transition buds from early flowering, as a trait; EF-Rb represents the reproductive buds from early flowering, as a trait; NF-Vb represents the vegetative buds from natural flowering, as a trait; NF-Tb represents the transition buds from natural flowering, as a trait; NF-Rb represents the reproductive buds from natural flowering, as a trait. A high degree of correlation between a specific module and the trait is indicated when the module name is highlighted in red. **b** Heatmaps showing genes in the modules that were significantly over-represented in EF-Vb

Correlated expression profiles imply that the genes operate in collaboration or in related pathways and that they contribute together to a given phenotype [63]. Our analysis identified 11 distinct modules (labelled with different colours), which are defined by major tree branches (Fig. S5). The number of genes in the modules ranged from 81 to 11,700. Four modules were highly expressed in one sample: MEdarkturquoise was highly associated with EF-Vb; MElightgreen was highly associated with NF-Vb; MEturquoise was highly associated with EF-Tb, and MEdarkgrey was highly associated with NF-Rb (Fig. 4a).

To explore the significance of the modules, correlations between the MEs and the three developmental periods were analysed. As the molecular regulation of floral transition starts before morphology changes occur, genes should have already changed in the vegetative stage to direct floral transition. The genes associated with floral transition should exhibit differential expression in Vb (Fig. 4b). Based on this principle, MEdarkturquoise was considered the main module of interest. In total, 1223 genes were included in the MEdarkturquoise module, among which 677 genes were known genes, and 564 genes were new genes (Table S6). To validate the accuracy of the transcriptome analysis results, 8 unigenes were selected for qRT-PCR confirmation. The expression profiles of the candidate unigenes revealed using qRT-PCR data were consistent with those derived from sequencing (Fig. S6).

**Fig. 5:**
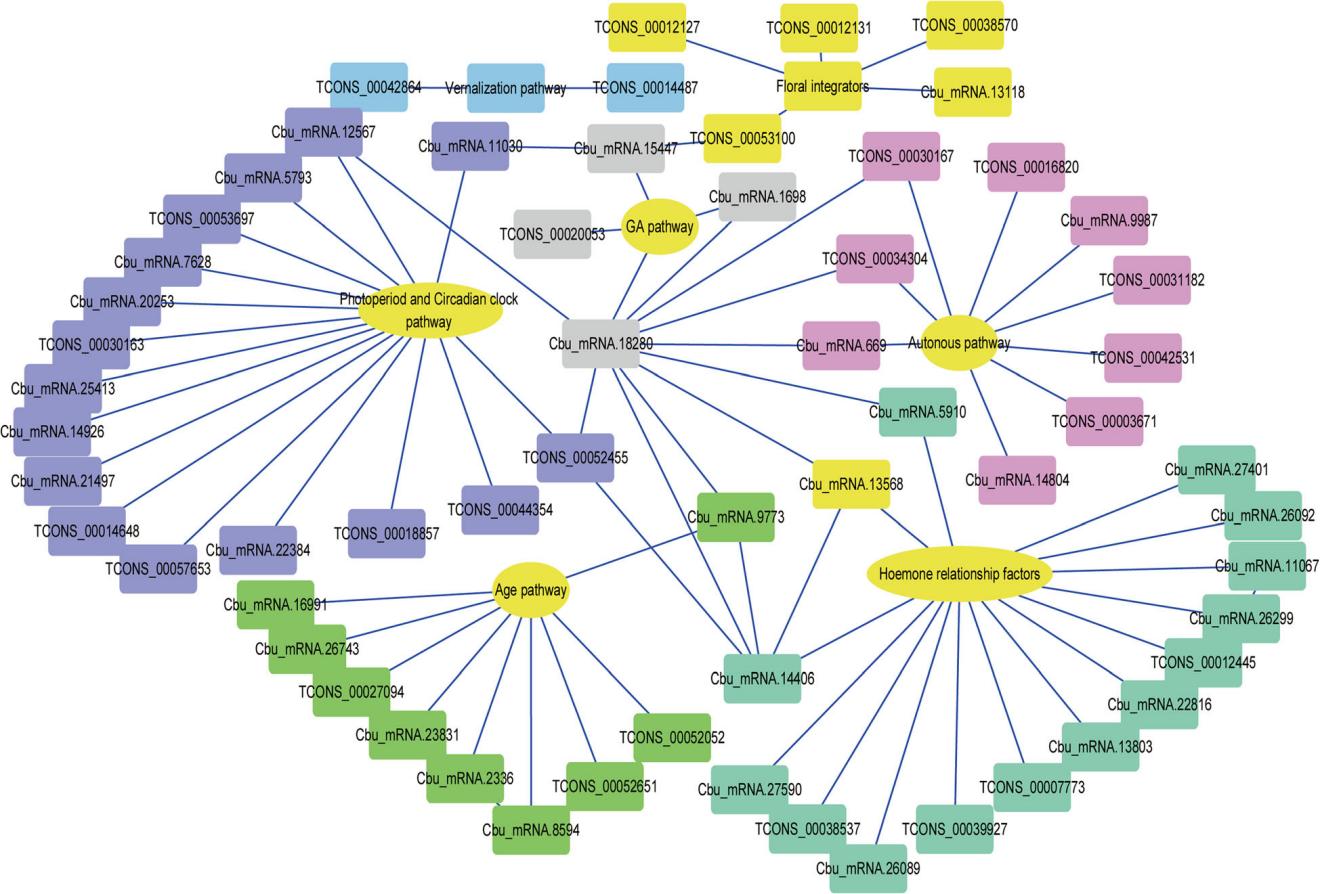
Correlation networks analysis of the top hub genes in MEdarkturquoise module. Yellow ovals represent the pathway names and rectangles of different colours represent individual genes. The genes in the purple shade were assigned to the photoperiod and circadian clock pathway. The genes in the green shade may belong to the age pathway. The genes in the brown shade may belong to the autonomous pathway. The genes in the grey shade may belong to the GA pathway. The genes in the blue shade may belong to the vernalisation pathway. The genes in the dark green shade may belong to the hormone relations factors. The genes in the yellow shade may belong to the floral integrator pathway

To study the relationship between these genes and floral transition more accurately, the top 10% of the genes were selected according to the correlation results. Sixty-one of these genes were annotated as hub genes involved in floral transition (Table 2, Table S7). The 61 hub genes were classed into the five floral regulation pathways, namely, the age pathway (Cbu.gene.9773 and Cbu.gene.16991, *SPL homologous genes*), autonomous pathway Cbu.gene.669, *FCA homologous gene*; Cbu.gene.14804, *FY homologous genes*), verbalization pathway (TCONS_00014487, *FRI homologous genes*), GA pathway (Cbu.gene.15447, *GA20ox homologous genes*; Cbu.gene.1698, *GA3ox homologous genes*) and photoperiod and circadian clock pathway (Cbu.gene.21497 *PIF homologous gene*; Cbu.gene.12567, *LUX homologous genes*; Cbu.gene.7628, CO *homologous genes*). In addition, several floral integrators, such as *SOC1* and *AP2*-like, and several hormone relation factors, including Cbu.gene.26092 and Cbu.gene.26299 (*ARF homologous genes*) (Fig. 5, Table 1), were detected. Subsequently, we analysed the regulatory network of the 61 hub genes in the MEturquoise module. Thirty-eight TFs were annotated from the regulatory network. Accordingly, the MIKC-MADS-box was shown to be highly related to floral transition [64–67].

**Table 1:** Summary of the 61 hub genes involving floral transition from the MEdarkturquoise module

Pathway	Gene ID		
Age pathway	TCONS_00052651	TCONS_00057653	TCONS_00027094
	Cbu_mRNA.23831	Cbu_mRNA.26743	TCONS_00052052
	Cbu_mRNA.9773	Cbu_mRNA.16991	Cbu_mRNA.2336
	Cbu_mRNA.8594		
Photoperiod and Circadian clock pathway	Cbu_mRNA.14926	TCONS_00052455	TCONS_00053697
	Cbu_mRNA.7628	Cbu_mRNA.5793	TCONS_00014648
	Cbu_mRNA.11030	TCONS_00018857	Cbu_mRNA.21497
	TCONS_00044354	TCONS_00030163	Cbu_mRNA.20253
	Cbu_mRNA.12567	Cbu_mRNA.22384	
Autonomous pathway	Cbu_mRNA.14804	TCONS_00016820	TCONS_00031182
	TCONS_00003671	TCONS_00034304	TCONS_00042531
	Cbu_mRNA.669	TCONS_00030167	Cbu_mRNA.9987
Verbalization pathway	TCONS_00014487	TCONS_00042864	
Hormome related pathway	Cbu_mRNA.1698	Cbu_mRNA.26092	Cbu_mRNA.27590
	Cbu_mRNA.15447	Cbu_mRNA.26089	Cbu_mRNA.11067
	Cbu_mRNA.13568	Cbu_mRNA.5910	Cbu_mRNA.27401
	Cbu_mRNA.14406	TCONS_00007773	TCONS_00038537
	TCONS_00039927	Cbu_mRNA.26299	Cbu_mRNA.13803
	TCONS_00012445	Cbu_mRNA.25413	TCONS_00012127
	Cbu_mRNA.22816		
Others	Cbu_mRNA.18280	Cbu_mRNA.9758	TCONS_00053100
	Cbu_mRNA.13118	TCONS_00020053	TCONS_00038570
	TCONS_00012131		

**Fig. 6:**
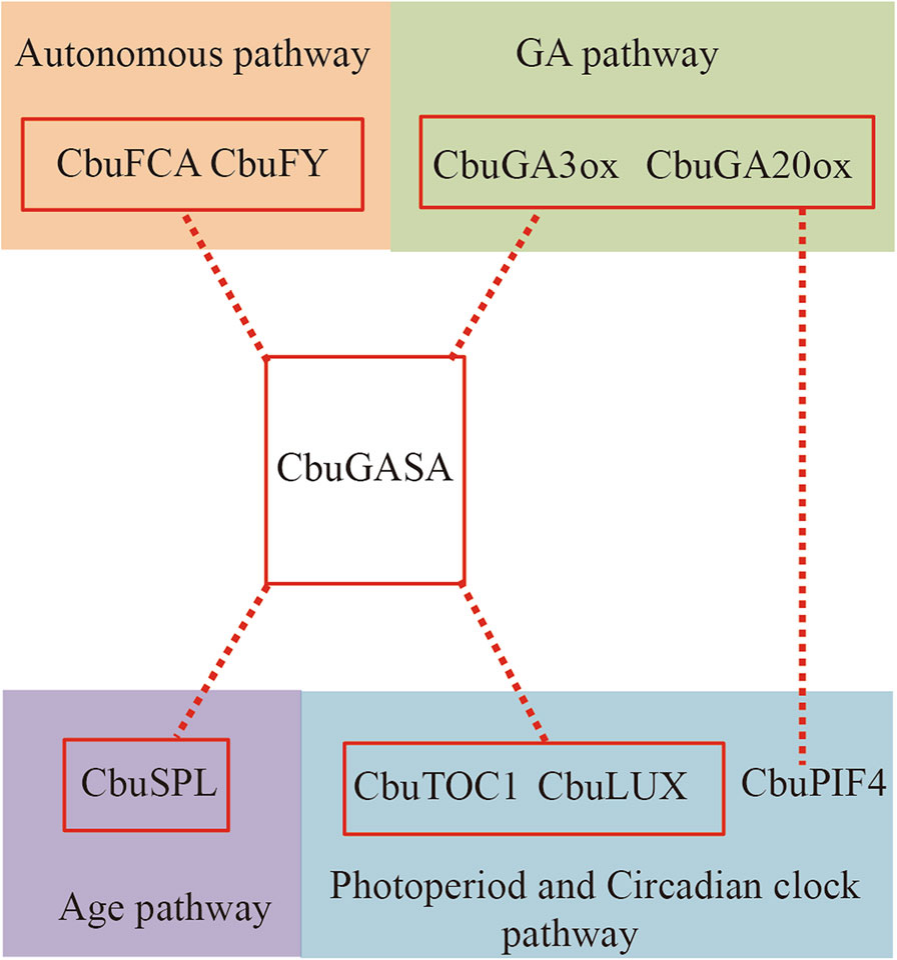
Hypothetical model for the networks of floral transition in *C. bungei*. Hypothetical model for the networks of floral transition in *C. bungei*. The red dotted lines show the new findings in this study, but relationships are uncertain and need further experiments

Interestingly, 10 out of the 61 hub genes had a close connection with Cbu.gene.18280, which was annotated as a *GASA homologous gene* (Fig. S7). According to WGCNA analysis, *GASA* was predicted to have high connectivity with *CbuSPL* (age pathway), *CbuFCA* and *CbuFY* (autonomous pathway), *CbuGA3ox* and *CbuG20ox* (GA pathway) and *CbuTOC1* and *CbuLUX* (photoperiod and circadian clock pathway). In addition, *CbuPIF4* (photoperiod pathway) and *Cbu*GA20ox (GA pathway) can affect the floral transition by promoting the expression of *CbuSOC1* (Fig. 6). However, floral transition is a very complicated process in *C. bungei* and needs to be further verified.

To verify the intersection results of GASA, we performed protein-protein interaction analysis (http://www.iitm.ac.in/bioinfo/PPA_Pred/prediction.html#). The dissociation constants (Kd), as well as on- and off-rates (k_on_ and k_off_) less than 10^− 9^, were set to predict protein binding. The protein interaction prediction results were highly consistent with the WGCNA results (Table 2).

**Table 2:** Protein interactions predicted with CbuGASA online

Gene ID	Annotation	Gene ID	Annotation	Delta G (binding free energy)/kcal/mol	Kd (dissociation constant)/M
Cbu_mRNA.18280	GASA	Cbu_mRNA.15447	GA20ox	−14.76	1.98E-12
Cbu_mRNA.18280	GASA	Cbu_mRNA.9773	SBP	−15.85	2.39E-12
Cbu_mRNA.18280	GASA	TCONS_00030167	FY	−15.40	5.08E-12
Cbu_mRNA.18280	GASA	TCONS_00018857	TOC	−14.57	2.08E-11
Cbu_mRNA.18280	GASA	Cbu_mRNA.12567	LUX	−14.56	2.09E-11
Cbu_mRNA.18280	GASA	Cbu_mRNA.16991	SBP	−13.40	1.48E-10
Cbu_mRNA.18280	GASA	Cbu_mRNA.20253	LUX	−13.18	2.17E-10
Cbu_mRNA.18280	GASA	Cbu_mRNA.669	FCA	−12.99	2.99E-10
Cbu_mRNA.18280	GASA	Cbu_mRNA.14804	FY	−12.66	5.19E-10
Cbu_mRNA.18280	GASA	Cbu_mRNA.1698	GA3ox	−12.16	1.21E-09
Cbu_mRNA.11030	PIF4	Cbu_mRNA.15447	GA20ox	−11.05	7.88E-09

To further study the correlation of *CbuGASA* and the 8 known hub genes (Table 2), we analysed the correlation coefficients of these mRNAs between the EF and NF samples during three developmental periods. Based on all trends, *CbuGASA* with 6 of 8 known hub genes (*CbuGA3ox*, *CbuSPLs*, *CbuLUXs*, *CbuFCA*) exhibited a positive correlation (r > 0.75) (Table S8). During the flowering process, the expression levels of mRNAs were significantly higher in the EF-Vbs than in the NF-Vbs, and gradually decreased with age. These results were consistent with the expression patterns of the homologous genes in *Arabidopsis*.

### Analysis of AS events from hub genes during floral transition development in *C. bungei*

**Fig. 7:**
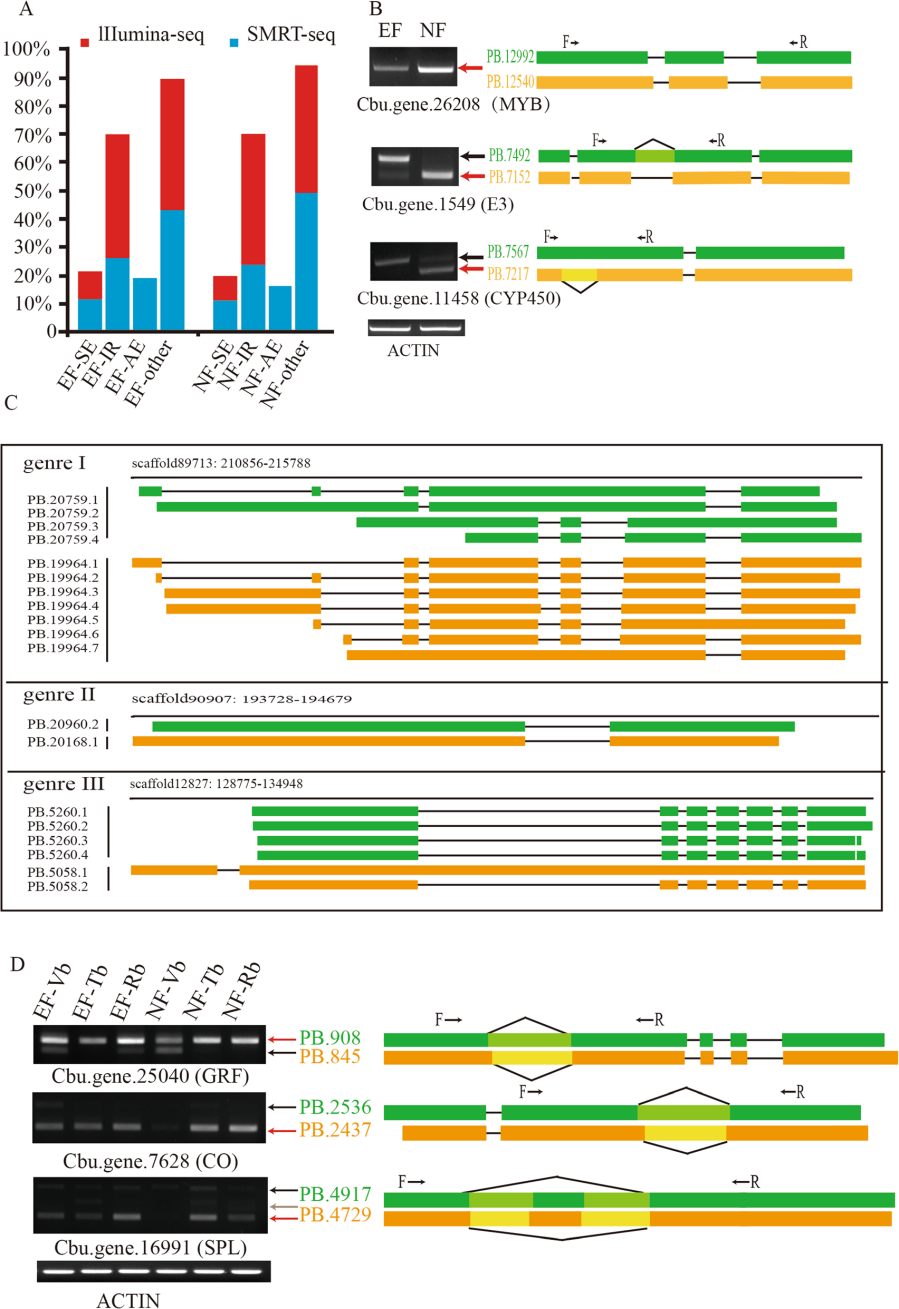
Characterization of AS events and validation of isoforms using reverse transcription polymerase chain reaction (RT)-PCR. **a** The proportion of different types of AS events detected by Illumina-seq or SMRT-seq in EF and NF. Classification of AS events: exon skipping (SE), intron retention (IR), and alternative exon (AE). **b** RT-PCR validation of AS events for five genes. Gel bands in each figure show PCR results in NF and EF. The transcript structure of each isoform is shown in the right panel. Boxes show exons in each transcript model. Green boxes show the isoforms from EF, and yellow boxes show the isoforms from NF. PCR primers (F, forward and R, reverse) are shown on the first isoform of each gene. **c** The number of genes belonging to the three genres. Genre-I contains the genes that have less isoforms in the EF variety than in the NF variety; genre-II contains the genes with similar numbers of isoforms in the EF and NF varieties. Genre-III contains the genes with less isoforms in the NF variety than in the EF variety. **d** The number isoforms of homologous genes in EF and NF*.* Gene names in the reference annotation (in parentheses) and corresponding names in the *C. bungei* genome are shown

Understanding AS events plays an extremely important role in understanding protein diversity [35, 68, 69]. On the basis of obtaining high-quality full-length isoforms, we performed a systematic analysis of AS in *C. bungei*. A total of 79,356 AS events from 25,662 mRNAs and 69,775 AS events from 25,046 mRNAs were detected in the two pools by SMRT. These AS events could be classified into five major types, namely, SE, IR, A5, A3, and AE [70]. However, no AE type was detected by Illumina (Fig. 7a). For both datasets, IR events showed the highest proportion in the EF and NF buds; SE events were the least frequent AS type in both the EF and NF buds. In addition, the percentage of the other more complicated AS types was greater in EF than in NF (Fig. 7a, Table S9). The AS event data from EF were compared with those from NF. A total of 23,851 and 22,936 common AS events from 9521 genes were detected in the EF and the NF buds. A total of 46,839 AS events associated with 15,526 genes were identified only in NF, and 55,505 AS events from 16,141 genes were detected only in EF (Table S10, Table S11). This result showed that SMRT is more accurate than Illumina for AS detection.

One of the most important features of SMRT is the ability to identify AS events by directly comparing isoforms of the same gene [33, 68, 71–74]. We randomly selected ten genes to evaluate the accuracy of AS events using RT-PCR (Table S12). The size of each amplified fragment was consistent with that of the predicted fragment (Fig. 7b, Fig. S8). These amplified fragments were then cloned for Sanger sequencing, and the amplified sequences were consistent with the SMRT data. These genes were divided into three groups according to the number of isoforms in EF and NF. The genes that had fewer isoforms in the EF than in NF were classified into genre-I, the genes with no obvious difference in the number of isoforms in EF vs NF were classified into genre-II, and the genes that had less isoforms in NF compared to EF were classified into genre-III (Fig. 7c). Further analysis of the AS events during floral transition was performed based on the SMRT data.

To evaluate the novel isoforms that may be involved in floral transition, we analysed the AS events of 200 genes (61 hub genes and their 10% target genes) in EF and NF. Among them, 41 genes were in genre-I, 36 genes were in genre-II and 28 genes were in genre-III. To study the specificity of the AS events in different stages, we verified the isoforms from three developmental periods in EF and NF by RT-PCR. For example, Cbu.gene.25040 (*GRF homologous gene*) was found in two isoforms in EF-Vb, NF-Vb and NF-Rb, and one Cbu.gene.25040 isoform was found in the rest of the buds. Cbu.gene.7628 (*CO homologous gene*) had different numbers of isoforms between EF-Vb and NF-Vb. Two Cbu.gene.7628 isoforms were found in EF-Vb, and one Cbu.gene.7628 isoform was found in NF-Vb. Cbu.gene.16991 (*SPL homologous gene*) had different numbers of isoforms between EF-Vb and NF-Vb. Two Cbu.gene.16991 isoforms were found in EF-Vb, and one Cbu.gene.16991 isoform was found in NF-Vb. In addition, there were different numbers of isoforms between Vb and Tb. There were three Cbu.gene.16991 isoforms in Tb from both EF and NF. (Fig. 7d, Fig. S9). The results showed that the genes in *C. bungei* exhibit divergent structures of isoform splicing in different bud stages.

## Discussion

Perennial ornamental woody plants have irreplaceable economic value. In this study, we analysed the data from three perspectives, namely, horizontal analysis (EF vs NF), vertical analysis (EF buds at different developmental stages) and WGCNA. We applied this strategy to the EF and NF samples, thereby enabling the correlation of specific expression information from transcriptional data to EF vegetative buds that formed during floral transition. Floral transition is a very complex regulatory system [4, 25, 75]. Multiple biological pathways, such as the age pathway, GA pathway, and photoperiod pathway, are involved in this process. The circadian rhythm pathway was significantly enriched in the whole floral transition cycle of EF compared with NF (Fig. 2c and Fig. 3b). This suggested that the circadian pathway might be critical for the early floral transition of EF. In addition, the photosynthesis pathway was mainly concentrated in Profile 7 (Fig. 2c), showing that this pathway gradually increased with the completion of floral transition. The circadian rhythm and photosynthesis pathways are important pathways that affect the reproductive transformation in Arabidopsis [18, 26, 76]. Circadian rhythm genes are regulated by the photosynthesis pathway and are used in related downstream photosynthesis pathways to regulate flowering. In the EF variety, the enrichment of circadian pathway genes may promote the activation of related downstream pathways, leading to early flowering.

TFs play important roles during development transition. Several TF families, such as *SBP*, *MIKC type MADS-BOX*, *ARF*, *bHLH*, and *G2-like*, were identified as floral transition-related in our study (Fig. 3a, Table S4). The floral transition process was regulated by multiple TFs in the EF variety, similar to the case of other plants. In this study, *SBP*, *ERF*, *bHLH*, *C3H* and *NAC* were the top 5 TFs by number in vegetative buds, and all *SBPs* were only enriched via upregulation in EF compared to NF in vegetative buds (Fig. 3c). In the EF/NF-Tb-UP groups, *SBP* was the top TF by number. Four *SBP* family members were identified in the 61 hub genes, which were determined by WGCNA (Fig. 5). *SBPs* are important floral transition regulators related with the age pathway [17, 77]. In Arabidopsis, *SPL*, which is negatively regulated by miR156, promotes floral transition by activating the expression of several other genes, such as *SOC*1, miR172, and *LFY* [5, 78–81]. Combined with the traits of the EF, the age pathway seems to be an important active pathway in floral transition, which implies that *SBPs* are important TFs affecting floral transition in EF.

A total of 1223 genes were included in the MEdarkturquoise module. To more preciselyanalyse the genes related to floral transition, we selected the top 10% of these genes according to connectivity. Sixty-one of 123 genes were annotated as specifically related to floral transition. These 61 genes were distributed into five pathways, i.e., the age pathway (e.g., *CbuSPL*), the photoperiod pathway (e.g., *CbuPIF, CbuCO* and *CbuLUX*), the autonomous pathway (e.g., *CbuFCA* and *CbuFY*), and the vernalisation pathway (e.g., *CbuFRI*), and *CbuGA20ox*, *CbuARF*, and *CbuERF* are hormone-related genes. These genes were highly expressed in EF-Vb. With the completion of floral transition, the expression of these genes decreased gradually, indicating that these genes may promote floral transition in *C. bungei*. In addition, several DEGs were annotated as *CbuSOC1* and *CbuAP2-LIKE*, which are critical genes in flowering. Most of our RNA-seq results were consistent with those in *Arabidopsis*, so the identified genes already had clear pathways in Arabidopsis. In addition to *SBP*, which regulates floral transition via the age pathway, several pathways were reflected in these data [82]. For example, *TOC1* and *LUX* are important factors in the three interlocking feedback loops in the circadian pathway, which mainly act on the upstream of the photoperiod pathway and ultimately regulate floral transition via the positive regulation of *CO* [8]. *FCA* and *FY* are autonomous pathway genes that have been studied in depth [83, 84]. *FCA* can inhibit the accumulation of *FLC*, which contains RNA binding proteins containing RNA recognition motifs (RRM) [8, 85]. FLC interacts with FY through the FCA WW domain, and the FCA/FY complex may negatively regulate *FLC* at the mRNA level [86]. *GA20ox* and *GA3ox* are critical for the synthesis of active GA [87]. The GA pathway influences floral transition mainly through two branches of active GA and *DELLA*. At the early stage of flower development, the active GA is involved in promoting the expression of *SOC1* and *LFY* and then the expression of downstream flowering genes. At the later stage of flower development, the active GA leads to the degradation of the *DELLA* protein and thus relieves the inhibition of flowering. Therefore, floral transition is guaranteed [87, 88]. Interestingly, several studies have revealed that DELLA regulates hypocotyl elongation by interacting with PIFs [75], contributing to floral transition by interacting with SPL [7], FD [10] and SOC1 [24, 62]. *SOC*1 plays an important role in regulating floral transition by integrating signals involved in all pathways, as well as interacting with many other genes to regulate floral transition [24]. However, all floral transition-related pathways still need to be further explored in *C. bungei*. These genes should be further studied to determine whether they are related to floral transition in the EF variety.

Furthermore, 41 highly connected gene pairs in the 61 hub genes were predicted by WGCNA (Fig. 5). Among these genes, Cbu.gene.18280 (*GASA homologous gene*) is particularly interesting. In this study, GASA had a strong interaction with GA20ox. *GA20ox* is an important gene for synthesizing GA3. These predictions were further verified by protein-protein interaction analysis online (Table 2). The *GASA* family is named for its GA3-induced expression in *Lycopersicon esculentum* [89–91]. In Arabidopsis, *GASA4* regulates floral meristem identity [90] and *GASA5* can extend flowering time by promoting the expression of *FLC* and *FT* [91]. GA plays an important role in the floral transition of plants, but more specific pathways need to be further studied [92]. We also found that CbuGASA was correlated with CbuFCA, CbuFY, CbuTOC1, CbuLUX and CbuSPL. The expression correlations of *CbuGASA* and its co-expressed genes suggest that *CbuGASA* is a positive regulator and involved in flowering regulation. These proteins/genes are important for floral transition by their respective pathways. This result provides important information for our subsequent research. Additional experiments need to be performed to verify the hypotheses about the role of CbuGASA in floral transition in *C. bungei*. To ensure more accurate transcriptome data, analysis of the genetic differences between the EF and NF varieties is one of the most important experiments we will undertake in future research.

In eukaryotes, AS greatly contributes to transcriptional diversity [33, 71, 74, 93]. AS produces multiple transcripts from a single gene and gives rise to proteins with various structures, subcellular localizations, stabilities and functions. AS has fundamental roles in a wide range of plant growth and development processes [41, 94–96]. Previous reports showed that isoforms have tissue specificity [93], and the ratio of the isoforms changes during the different growth periods [44]. For example, flowering integrator *FT* is also subjected to AS events in temperate grasses. The ratio of the two AS evens of *FT* was progressively reduced during development, indicating that one of the two AS events is regulated by endogenous cues rather than an external cue for flowering. In our study, similar results were found. For example, the key genes in the floral transition of *C. bungei*, *CbuCO* (Cbu.gene.7628) and *CbuSPL* (Cbu.gene.16991) are also subjected to AS during the flowering process. In Vb of EF and NF, the ratio of AS in Vb (2:1) was higher than that of the other two periods of EF and NF (1:1). In addition, *CbuSPL* had more isoforms in Tb than in Vb in both the EF and NF buds. The above results imply that AS events may have an important role in floral transition in *C. bungei*. These observations indicate that AS greatly increases the complexity of gene transcription in *C. bungei*, and more experiments are needed to test this hypothesis. For example, we should not only consider how genes are transcribed in development periods but also investigate the functional differences of homologous genes in *C. bungei*. The multi-omics data were integrated to explore the floral transition mechanism in *C. bungei*.

## Conclusions

This study expands our view of the transcriptomes of *C. bungei* during floral transition. A number of DEGs were detected in vegetative to reproductive growth buds following WGCNA analysis. These genes belonged to pathways that collectively regulate floral transition, and the results enhance our understanding of gene regulation during floral transition in perennial woody plants. Furthermore, SMRT analysis provided the first insights into AS events in *C. bungei*. Frame usage in the same transcript further increases the genetic complexity of *C. bungei*. These results will facilitate future functional genomics studies.

## Methods

### Plant material and experimental procedures

*C. bungei* is a perennial plant that flowers after 5 years of forestation (http://www.forestry.gov.cn/). A natural EF variety that flowers after 1-year forestation was found in Henan province, China (Fig. 1a). This EF has been applied to produce new varieties of *C. bungei*, which are named ‘bairihua’. From 28 February to 31 March 2016, we collected the buds from the first round to the axillary buds of EF and NF varieties at an interval of every 1–2 days. All samples were collected from 9:00–12:00 and transferred immediately to liquid nitrogen for SMRT- and Illumina-based RNA sequencing and reverse transcription-polymerase chain reaction (RT-PCR). Using paraffin section analysis, vegetative buds (Vb), transition buds (Tb) and reproduction buds (Rb) were identified for transcriptome sequencing.

### Library preparation and PacBio sequencing

To construct libraries for Pacific Biosciences (PacBio) sequencing, equal amounts of EF and NF buds from each stage (vegetative stage, transition stage and reproduction stage) were pooled together. Total RNA from the NF buds for three periods, NF-Vb, NF-Tb and NF-Rb, was mixed to provide the ‘NF’ sample for comparison with the EF sample. For EF buds, total RNA from three periods, EF-Vb, EF-Tb and EF-Rb, was mixed to provide the ‘EF’ sample. The two mixed RNA samples from buds were reverse transcribed using the SMRT _er_® PCR cDNA Synthesis Kit. PCR amplification was carried out using the KAPA HiFi PCR Kit. The product was separated by agarose gel-based size selection into cDNA fractions 1–2 kb, 2–3 kb and > 3 kb in length. These SMRT libraries were generated using the PacBio 1.0 Template Preparation Kit (Menlo Park, CA, USA, part #001–322-716) according to the standard protocol. The 1–2 kb library was sequenced using five SMRT cells, and the other two libraries were sequenced using four SMART cells. The cDNA products were purified for library construction using the SMRTbell Template Prep Kit 1.0. Libraries were sequenced using P6C4 polymerase (PacBio, P/N 100–372-700) and chemistry on the PacBio RS II platform with 240-min movie times. Each size fraction for each sample was run through the Iso-Seq pipeline included in the SMRT analysis software package individually. First, ROIs (previously known as circular consensus sequences) were generated using the minimum filtering requirement of 0 or more passes of the insert and a minimum read quality of 75. This requirement allows for the highest yield going into the subsequent steps, while creating higher-accuracy consensus sequences when possible. The pipeline then classified the ROIs as full-length non-chimeric or non-full-length reads. Full-length reads with lengths of at least 300 bp were determined by detecting poly(A) tails, 5′ primers and 3′ primers. All full-length reads were aligned to the *C. bungei* genome using G_MAP_ software.

### Illumina RNA sequencing of EF and NF buds

The samples stages were used to construct 18 libraries for Illumina-based RNA sequencing, which were named NF-Vb, NF-Tb, NF-Rb, EF-Vb, EF-Tb and EF-Rb. Each stage had three biological replicates. Total samples were sent to the Beijing Genomics Institute for strand-specific library construction and sequencing on an Illumina HiSeq 4000 platform. In total, 13G of 150-bp paired-end reads were generated. Raw sequence data of the libraries for differentially expressed gene (DEG) profiling analysis were filtered to remove reads containing adapters, reads with an unknown nucleotide content exceeding 10% unknown nucleotides, and reads with a low-quality base (value <=5) content greater than 50%. Clean reads were mapped into the transcriptome reference database using SOAP software. No more than 2 mismatched bases were permitted, and unique mapped reads were obtained. Fragments per kilobase of exon per million fragments mapped (FPKM) was used to obtain the relative expression levels. A differential expression analysis of the two groups was performed using the DESeq package. The resulting *P*-values were adjusted using Benjamini and Hochberg’s approach for controlling the false discovery rate. DEGs with a |fold change| > = 1.2 and an FDR < 0.05 were identified between each comparison. The DEG expression data υ (from Vb to Tb and Tb to Rb) were normalized to 0, log_2_^(Tb/Vb),^ and log_2_^(Rb/Vb)^. DEGs were clustered using the Short Time-series Expression Miner (STEM). To analyse gene co-expression patterns based on mRNA profiles in buds from the EF and NF varieties, WGCNA was performed according to Langfelder and Horvath (2008) [97]. Here, we chose a power of four so that the resulting networks exhibited approximate scale-free topology (model fitting index R2 = 0.80). The resulting gene dendrogram was used for module detection using the Dynamic Tree Cut method (minimum module size = 50) [63]. Co-regulated genes are grouped into modules based on the corresponding genes’ information. In addition, the intra-modular hub genes refer to highly connected genes in a module. They can be determined by calculating the Pearson correlation between the expression level and the module eigengene. In this study, the top 10% of genes with high correlation were considered as hub genes for a given module. Finally, the modules of interest were input into Cytoscape to determine network information [44].

With the Gene Ontology (GO; http://www.geneontology.org) and Kyoto Encyclopedia of Genes and Genomes (KEGG; https://www.kegg.jp/) pathway annotation results, we classified mRNAs according to official classifications, and we also performed GO and pathway functional enrichment using Phyper, a function of R software. The parameters for Phyper were set as *P*-values 0.05 after Bonferroni correction. The phyper function in R was used to analyse the P-value for each function theme:

$$ \mathrm{P}=1\hbox{-} \sum \limits_{i=0}^{m-1}\frac{\left(\begin{array}{c}M\\ {}i\end{array}\right)\left(\begin{array}{c}N-M\\ {}n-i\end{array}\right)}{\left(\begin{array}{c}N\\ {}n\end{array}\right)} $$

Smaller *P*-values were associated with greater enrichment of the candidate genes in a given function theme (https://en.wikipedia.org/wiki/Hypergeometric_distribution). The generation of Venn diagrams and hierarchical clustering heat maps in this study were conducted using the gmodels, Venn diagram and Pheatmap packages in R (https://www.r-project.org/) based on the gene list and the gene expression levels for each type.

### Pipeline for isoform sequencing analysis

To classify AS events, the tool AStalavista was employed using the raw.gtf file assembled from the Illumina RNA-seq and SMRT sequencing data. AS analysis was conducted using SpliceGrapher by converting the detected splice isoforms into splice graphs. Introns fully subsumed by an exon were labelled as retained. Overlapping exons that differed at their 5′ or 3′ splice junctions were considered alternative 5′ or 3′ splicing events, respectively. Finally, exons absent in other isoforms were considered exon skipping events.

MATS was used to call the differentially spliced events between the EF and NF samples at the three development periods, using the aligned.bam files as input with default settings. For comparison, the merged.gtf file derived from the SMRT and new Illumina data was used as a reference. The examined events included skipped exon (SE), alternative 5′ splice site (A5), alternative 3′ splice site (A3), alternative exons (AE), and retained intron (IR) events.

### RNA extraction, quantification, and RT-PCR

Total RNA was extracted using RNA Reagent (RN38; Aidlab Biotechnology, Beijing, China) according to the manufacturer’s protocol and treated with RNase-free DNase I (Takara, Dalian, China) to remove genomic DNA contamination. First-strand cDNA was generated from 1 μg of total RNA isolated from buds using the superscript first-strand synthesis system (Invitrogen, USA). The specific primers were designed using Primer 3plus and synthesized by Majorbiogene Co., Ltd. (Beijing, China). The melting temperature of the primers was 60 °C, and the amplicon lengths were 100–200 bp. Real-time qRT-PCR was performed on a Roche LightCycle 480 Real-Time PCR System (Roche Applied Science, Germany) using a SYBR Premix Ex Taq™ Kit (TaKaRa, Dalian, China) according to the manufacturer’s instructions. *Cbu-actin* was used as an internal control, and each reaction was conducted in triplicate [31]. All the primers are shown in Table S12. Each reaction was performed in a total reaction mixture volume of 20 μL containing 2 μL of first-strand cDNA as template. The amplification program was as follows: 3 min at 95 °C and 30 cycles of 15 s at 95 °C, 30 s at 60 °C, 1 min at 72 °C and 10 min 72 °C. Each reaction was performed with three replicates. The expression levels of candidate genes were determined by CT values and calculated by the 2^−△△Ct^ method. We test the correlation of expression (CEG) between mRNAs by using the Pearson correlation coefficient. The Pearson correlation coefficient was calculated by COR() using the average relative expression of three replicates in R [98].

### Transcription factor prediction and protein and protein interaction analysis

We found the ORF of each DEG by using getorf (version: EMBOSS: 6.5.7.0, parameters: -minsize 150, //www.bioinformatics.nl/cgi-bin/emboss/help/getorf). We aligned ORFs to transcription factor (TF) domains from PlntfDB (http://plntfdb.bio.uni-potsdam.de/v3.0/) by using hmmsearch (http://hmmer.org). We used DIAMOND (version: v0.8.31, parameters: --evalue le-5 –outfmt 6 –max-target-seqs 1 —more-sensitive, https://github.com/bbuchfink/diamond) to map the DEGs to the STRING (version: v10, http://string-db.org/) database to obtain interactions between DEG-encoded proteins using homology with known proteins.

## Supplementary information

**Additional file 1: Fig. S1.** Read length and quality in EF. (A) Read length distribution in the 1–2 kb library. (B) Read quality distribution in the 2–3 kb library. (C) Read length distribution in the above 3 kb library.

**Additional file 2: Fig. S2.** Read length and quality in NF. (A) Read length distribution in the 1–2 kb library. (B) Read quality distribution in the 2–3 kb library. (C) Read quality distribution in the 3–6 kb library. (D) Read quality distribution in the above 6 kb library.

**Additional file 3: Fig. S3**. Characterization of the *C. bungei* transcriptome by SMRT-seq. (A) Distribution of transcript lengths from different sequencing platforms. (B) Venn diagram showing the common and unique annotated genes detected by SMRT and Illumina. (C) Distribution of the number of APA sites per gene.

**Additional file 4: Fig. S4.** Analysis of differential gene expression of the NF. (A) The 8 significant expression profiles of NF. (B) Partial KEGG pathways associated with the NF.

**Additional file 5: Fig. S5.** Hierarchical cluster tree showing all modules.

**Additional file 6: Fig. S6**. qRT-PCR validation of mRNA for 14 genes.

**Additional file 7: Fig. S7**. qRT-PCR validation of mRNA for 10 genes.

**Additional file 8: Fig. S8**. RT-PCR validation of AS events for 5 genes. This figure is supplemental to Fig. 7b.

**Additional file 9: Fig. S9**. RT-PCR validation of AS events in three development periods. This figure is supplemental to Fig. 7d.

**Additional file 10: Table S1.** Summary of Illumina short reads.

**Additional file 11: Table S2**. Statistics of the SMRT sequencing data.

**Additional file 12: Table S3**. Top 20 KEGG pathways significantly enriched with DEGs during floral transition in EF.

**Additional file 13: Table S4**. All predicted transcription factors in EF during floral transition.

**Additional file 14: Table S5**. Top 20 KEGG pathways significantly enriched with DEGs in three different stages between EF and NF.

**Additional file 15: Table S6**. Summary of all genes from the MEdarkturquoise module.

**Additional file 16: Table S7**. Summary of DEGs classified into five pathways.

**Additional file 17: Table S8**. The correlation coefficients of expression of mRNAs between the EF and NF during three developmental periods. r is the correlation coefficient of expression of the mRNAs.

**Additional file 18: Table S9**. Summary of identification of splicing junctions using NGS and SMRT data.

**Additional file 19: Table S10**. Summary of AS events from DEGs in EF.

**Additional file 20: Table S11**. Summary of AS events from DEGs in NF.

**Additional file 21: Table S12**. Summary of primers used in this study.

## Data Availability

The sequencing data have been submitted to the Sequence Read Archive (SRA) at the National Centre for Biotechnology Information. The accession number is PRJNA414524. The data supporting the conclusions of the article have been uploaded as additional files.

## Abbreviations

NGS: Short-read next-generation sequencing
SMRT: Single-molecule real-time
EF: Early flowering
NF: Normal flowering
WGCNA: Weighted Gene Co-expression Network Analysis
AS: Alternative splicing
SAM: Shoot apical meristem (SAM)
SPL: Squamosa-promoter binding protein-like
TOC: Timing of cab expression 1
LUX: Luxarrhythmo
PIF: Phytochrome interacting factor
CO: Constans
FRI: Frigida
GA20ox: GA20oxidases
GA3ox: GA3oxidases
SOC1: Suppressor of overexpression of constans 1
Vb: Vegetative bud
Tb: Transition bud
Rb: Reproductive bud
APA: Alternative polyadenylation
STEM: Short Time-series Expression Miner
DEG: Differentially expressed gene
GASA: GA-Stimulated in Arabidopsis
HSP: Heat shock protein
NAC: Nascent polypeptide associated complex
MYB: v-myb avian myeloblastosis viral oncogene homolog
bHLH: basic helix-loop-helix protein
FPKM: Fragments per kilobase million
AP2: Apetala 2
Kd: Dissociation constants
SE: Exon Skipping
IR: Intron Retention
A5: Alternative 5′ Splice Site
A3: Alternative 3′ Splice Site
AE: Alternative Exon
GRF: Growth regulating factor
ARF: Auxin- responsive factor
G2-like: Golden2-Like
ERF: Ethylene-responsive factor
LFY: Leafy
FD: Flowering locus D
