# Supplementary figures and images for: Multi-omics sequencing provides insight into floral transition in Catalpa bungei. C.A. Mey
Source: BMC Genomics. 2020 Jul 22;21:508. doi: 10.1186/s12864-020-06918-y (PMC7376858; doi:10.1186/s12864-020-06918-y)

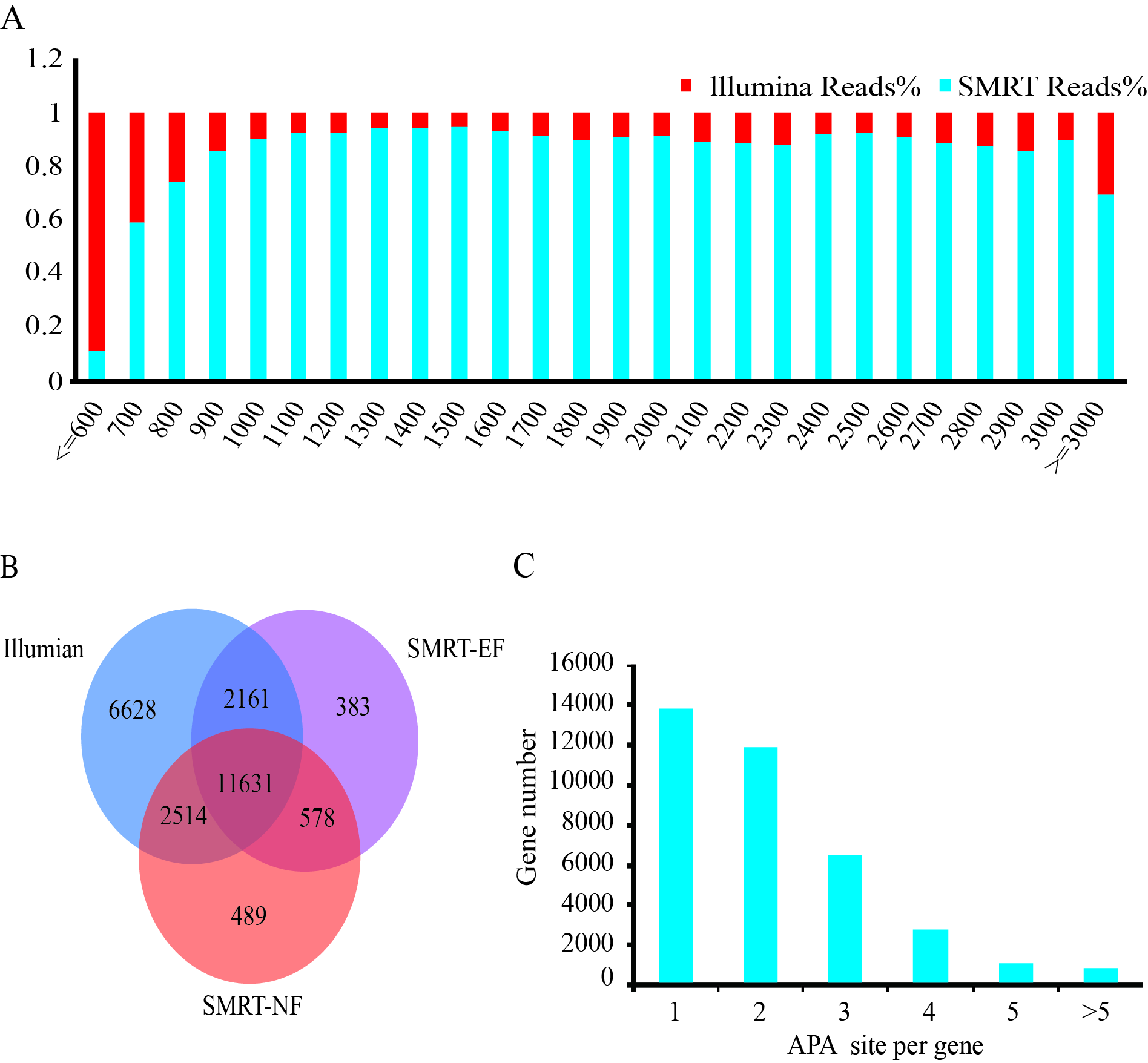

Supplement: Supplementary file 3 — Additional file 3: Fig. S3. Characterization of the C. bungei transcriptome by SMRT-seq. (A) Distribution of transcript lengths from different sequencing platforms. (B) Venn diagram showing the common and unique annotated genes detected by SMRT and Illumina. (C) Distribution of the number of APA sites per gene. [file 12864_2020_6918_MOESM3_ESM.tif]

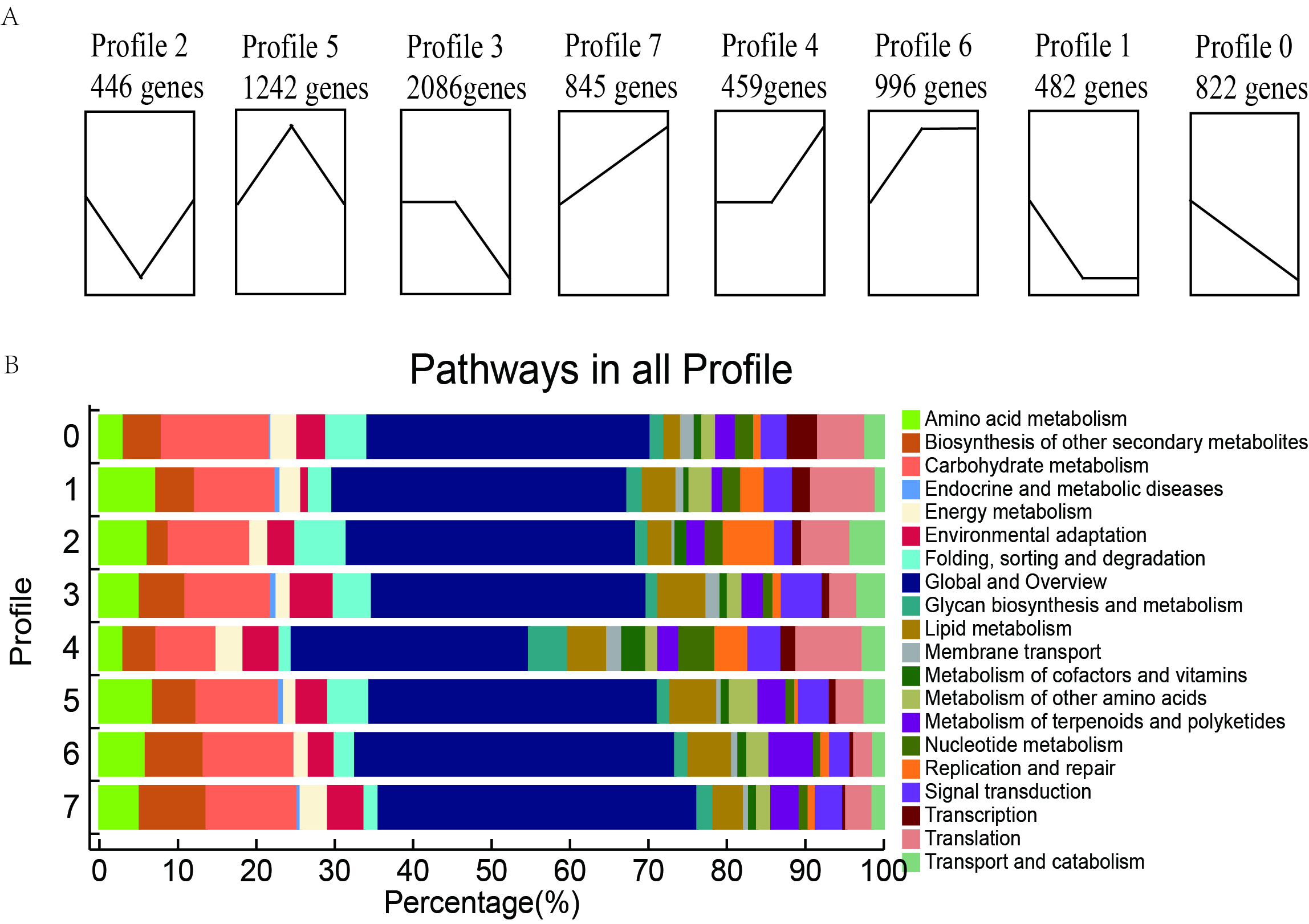

Supplement: Supplementary file 4 — Additional file 4: Fig. S4. Analysis of differential gene expression of the NF. (A) The 8 significant expression profiles of NF. (B) Partial KEGG pathways associated with the NF. [file 12864_2020_6918_MOESM4_ESM.tif]

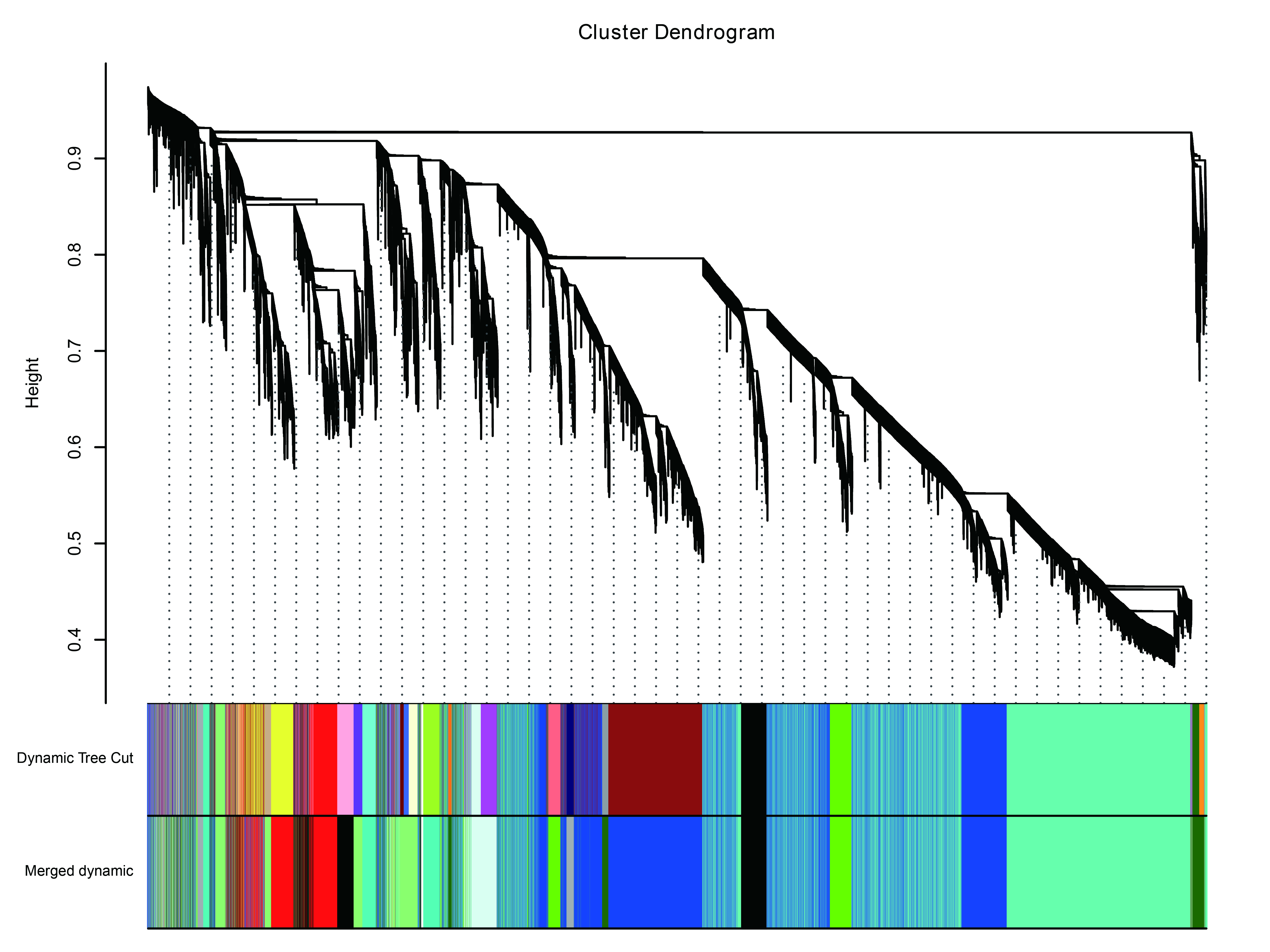

Supplement: Supplementary file 5 — Additional file 5: Fig. S5. Hierarchical cluster tree showing all modules. [file 12864_2020_6918_MOESM5_ESM.tif]

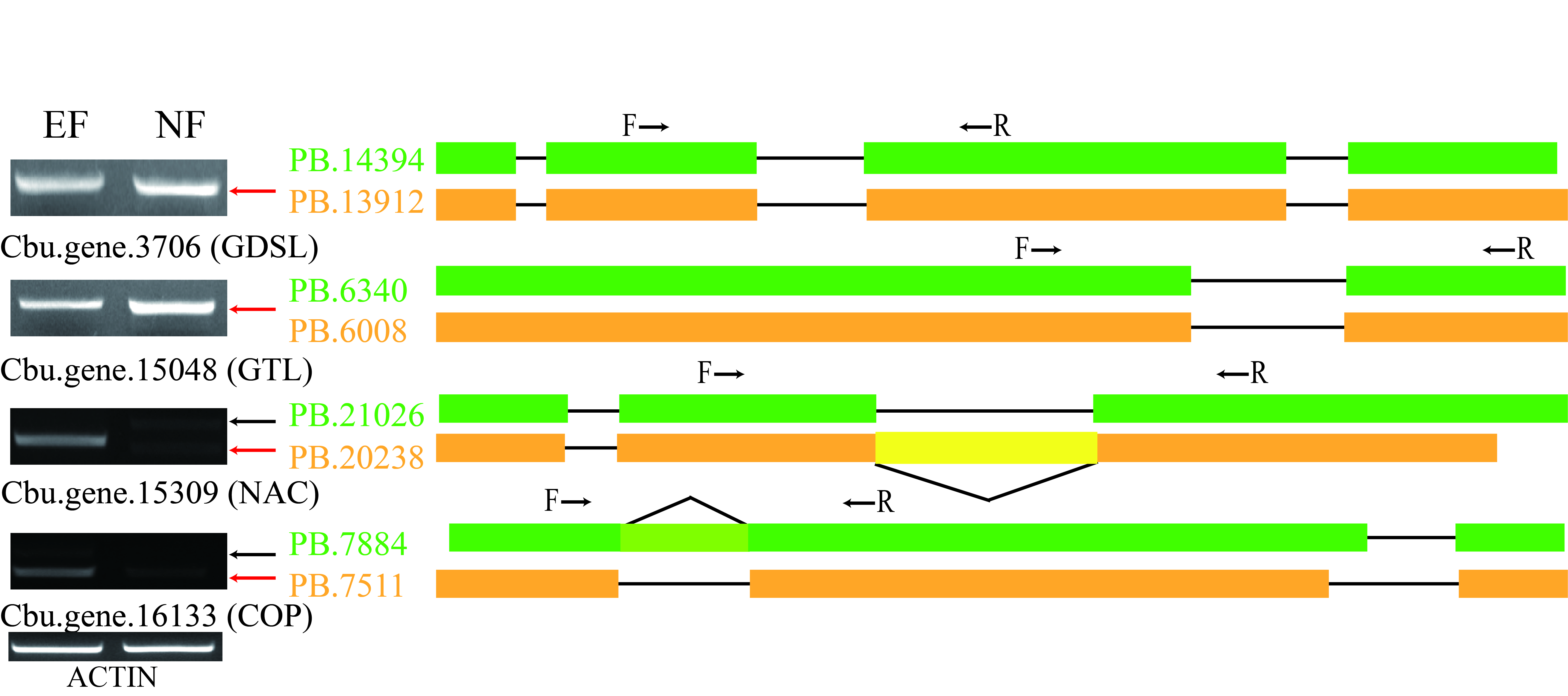

Supplement: Supplementary file 8 — Additional file 8: Fig. S8. RT-PCR validation of AS events for 5 genes. This figure is supplemental to Fig. 7b. [file 12864_2020_6918_MOESM8_ESM.tif]

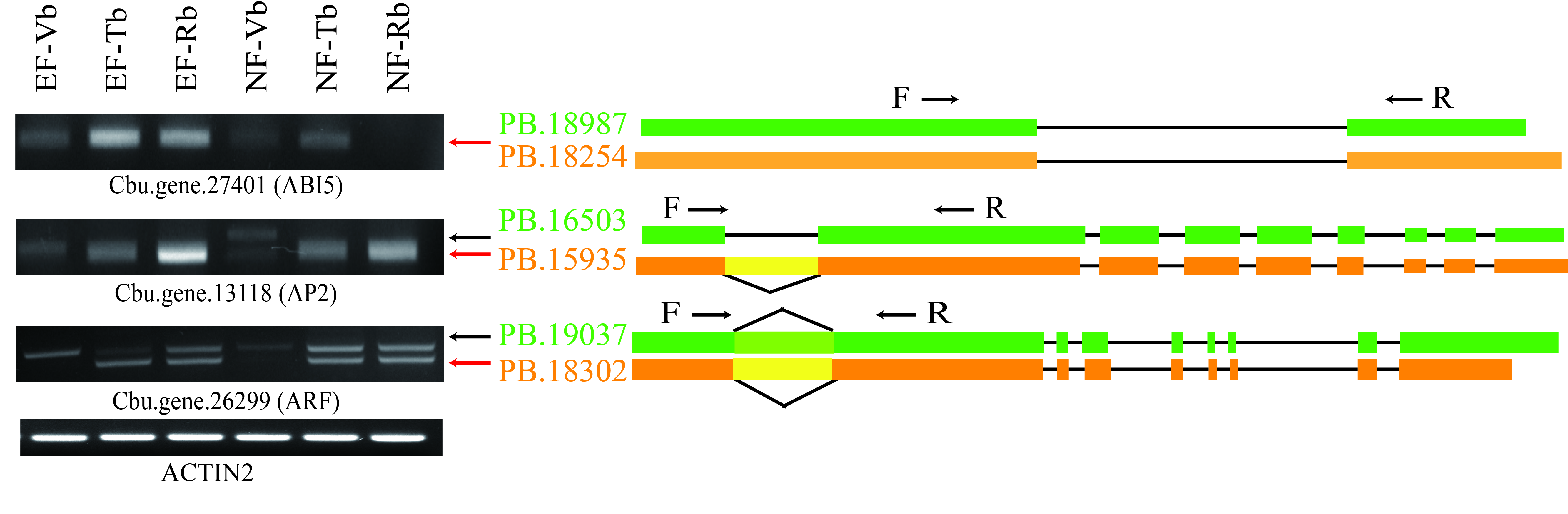

Supplement: Supplementary file 9 — Additional file 9: Fig. S9. RT-PCR validation of AS events in three development periods. This figure is supplemental to Fig. 7d. [file 12864_2020_6918_MOESM9_ESM.tif]
