# Supplementary material for: Multi-omics sequencing provides insight into floral transition in Catalpa bungei. C.A. Mey
Source: BMC Genomics. 2020 Jul 22;21:508. doi: 10.1186/s12864-020-06918-y (PMC7376858; doi:10.1186/s12864-020-06918-y)
Supplement: Supplementary file 11 — Additional file 11: Table S2. Statistics of the SMRT sequencing data. [file 12864_2020_6918_MOESM11_ESM.docx]

| Library | 1-2 kb | 2-3 kb | >=3 kb |
| --- | --- | --- | --- |
| EF |  |  |  |
| No.of SMRT cells | 4 | 4 | 5 |
| ReadsOfInsert | 142240 | 138258 | 111800 |
| Read Bases of Insert (bp) | 222222224 | 375971505 | 427836826 |
| Mean Read of length Insert (bp) | 1562 | 2719 | 3826 |
| Mean Read quality of insert | 0.94 | 0.91 | 0.89 |
| Mean number of passes | 13 | 8 | 7 |
| poly-A reads | 107,594(75.64%) | 92.099(66.61%) | 63,702(41.07%) |
| Full-length non-chimeric reads | 95532 | 75202 | 45917 |
| Full-length non-chimeric read length | 1490 | 2568 | 3719 |
| NF |  |  |  |
| No.of SMRT cells | 6 | 4 | 6 |
| ReadsOfInsert | 216358 | 153048 | 92626 |
| Read Bases of Insert (bp) | 279425623 | 444682022 | 327190309 |
| Mean Read of length Insert (bp) | 1291 | 2905 | 7888 |
| Mean Read quality of insert | 0.94 | 0.91 | 0.89 |
| Mean number of passes | 14 | 8 | 5 |
| poly-A reads | 165,939(76.7%) | 101,517(66.33%) | 66.015(67.71%) |
| Full-length non-chimeric reads | 141,23 | 81385 | 41582 |
| Full-length non-chimeric read length | 1179 | 2065 | 4571 |

**Table S2 Statistics of the SMRT sequencing data**
